# RNA Virus Reverse Genetics and Vaccine Design

**DOI:** 10.3390/v6072531

**Published:** 2014-06-25

**Authors:** Christopher C. Stobart, Martin L. Moore

**Affiliations:** 1 Department of Pediatrics, Emory University School of Medicine, Atlanta, GA 30322, USA; E-Mail: c.c.stobart@emory.edu; 2 Children’s Healthcare of Atlanta, Atlanta, GA 30322, USA

**Keywords:** RNA virus, reverse genetics, vaccines

## Abstract

RNA viruses are capable of rapid spread and severe or potentially lethal disease in both animals and humans. The development of reverse genetics systems for manipulation and study of RNA virus genomes has provided platforms for designing and optimizing viral mutants for vaccine development. Here, we review the impact of RNA virus reverse genetics systems on past and current efforts to design effective and safe viral therapeutics and vaccines.

## 1. Introduction

Vaccines remain one of the greatest accomplishments of human ingenuity, scientific endeavor, and the combined global efforts of the public health community. The rates of incidence and mortality associated with infection by RNA viruses such as polio, measles, mumps and rubella have declined by greater than 95% compared to pre-vaccination rates [1]. Though highly successful in the past, conventional approaches to RNA virus vaccine development, such as live-attenuation through passaging (forward genetics) or inactivation, may be less efficient for generating good candidates than rational targeted mutagenesis (reverse genetics). Advancements in recombinant DNA technology and virus reverse genetics have provided key critical insights into the replication and pathogenesis of RNA viruses and facilitate vaccine development through targeted modifications and directed attenuation. The advent of reverse genetics and molecular engineering of viruses has transformed the field of virology by permitting study of targeted genetic changes in virus genomes. In 1981, the first infectious RNA virus clone was isolated from cDNA to generate poliovirus [2]. Since then, reverse genetics technology and recombinant virus design has been employed to generate reverse genetic clones representing all major virus families. In addition, these techniques and approaches have now become the focus of new efforts to design vaccines that incorporate specific changes in either component-based or virus-based systems to induce lasting immunity in the host without health risks or deleterious effects. 

Since the development of effective live-attenuated vaccines, new vaccine preparations utilizing well-established vectors, expression of specific viral proteins or components (subunit vaccines), and development of virus-like particles (VLPs), have continued to shape the domain of vaccine discovery and development. The challenge of establishing a safe, immunogenic platform, which induces lasting immunity in the context of a wide variety of viral systems, has resulted in remarkable creativity and variability in the approaches employed. Use of replicating viruses in vaccines, such as live-attenuated or chimeric vector-based platforms, have the benefits of high immunogenicity, lower costs, and ease to transport and administer. Yet, these viruses have the potential to revert to more pathogenic phenotypes and may be under-attenuated in immunocompromised hosts. Conversely, component, subunit, or killed pathogen vaccines have the benefits of generally being safer and can be used to display the most immunogenic antigens. However, the costs, time of development, and weaker induced immune responses present their own challenges to design and implementation. In this review, we describe RNA virus reverse genetics systems and provide an overview of current efforts to use reverse genetics technology in the development of safe and effective vaccines.

## 2. RNA Virus Biology and Reverse Genetic Infectious Clone Design

**Figure 1:**
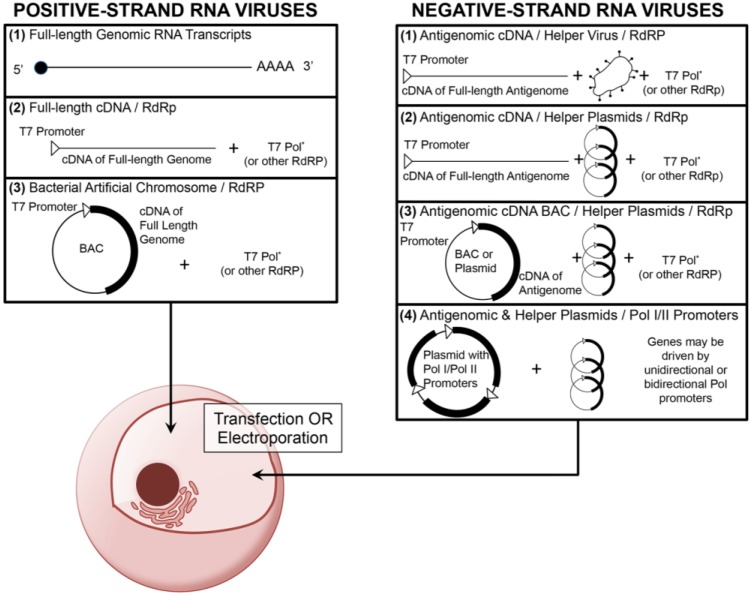
An overview of common reverse genetic platforms for the recovery of positive- and negative-strand RNA viruses. Most positive-strand RNA virus reverse genetic platforms consist of either direct introduction of full-length copies of the viral genome (which have been transcribed *in vitro*) or introduction of either a linear or plasmid‑associated (bacterial artificial chromosome, BAC) cDNA of the full-length genome in combination with an RdRp (such as T7 polymerase). Negative-strand RNA virus reverse genetic platforms often involve transfection or electroporation of genomic or more commonly subgenomic cDNA into permissive cells in combination with either a helper virus or helper plasmids, all of which driven by a RdRp. Some negative-strand RNA systems employ host polymerase I (Pol I) and II (Pol II) promoters to drive viral RNA synthesis and mRNA production. In both positive- and negative-strand reverse genetics systems, the RdRp (*) is typically constitutively or transiently expressed in the permissive cell type.

Current RNA virus reverse genetics systems make use of multiple common features of RNA virus biology. First, RNA viruses generate genomic copies through the activity of a viral RNA-dependent RNA polymerase (RdRp). In addition, nearly all RNA virus replication strategies are independent from the host cell nucleus and instead reside in the cytoplasm. For positive-strand RNA viruses, such as poliovirus, immediately after entry and uncoating, the genomic RNA is directly translated by host ribosomes to generate viral protein products. Since the virus rarely needs to package additional non‑structural proteins in the virion, most positive-sense RNA virus reverse genetics systems largely focus on delivery of either transcribed genomic RNA into the cell cytoplasm or delivery of cDNA under the control of a viral transcription promoter such as T7 or CMV (Figure 1) [3,4,5,6,7,8,9,10]. However, negative-strand and double-strand RNA virus reverse genetic systems often require the use of additional helper constructs to introduce the RdRp and other essential proteins to initiate genomic replication. A recent alternative approach, which has been employed in the field of influenza research, is to synthesize viral RNA and drive mRNA production through the activity of the host polymerases such as Pol I and Pol II [11,12]. This approach has simplified the logistics of plasmid transfection and increased the efficacy of recombinant virus recovery. A similar approach has been successfully employed for recovery of an arenavirus, lymphocytic choriomenigitis virus (LCMV) [13]. One additional platform that has been employed for RNA virus infectious clone generation is the bacterial artificial chromosome (BAC). BAC constructs are single-copy DNA plasmids based on the F-plasmid of bacteria, which are genetically stable in *E. coli* and permit the insertion of large DNA fragments to be transcribed under the control of a transcriptional promoter such as T7. BAC constructs have been employed for many years in the recovery of large DNA viruses. However, the genetic stability of these constructs has led to their use in recombinant RNA virus platforms. Several positive-strand RNA viruses and one negative-strand RNA virus have used BACs as a platform for reverse genetic design (Table 1). Since most RNA virus replication strategies are segregated from the host genome and replication machinery, few RNA viruses modify host gene expression and cause oncogenesis. As a result, RNA reverse genetic systems do not typically have to account for the potential transformation of host cells. Lastly, most RNA viruses are limited to small genome sizes with the majority smaller than 15 kb due to reduced genomic stability and the lowered fidelity of the viral RdRp. Subsequently, reverse genetic approaches often employ the use of cDNA genetic clones for greater versatility in manipulation and modification of the virus genome. 

**Table 1:** BAC-based reverse genetics platforms for recovery of recombinant RNA viruses.

Virus	Family	Genome	Genome Size	Year	Reference
Transmissible gastroenteritis virus (TGEV)	Coronaviridae	+ssRNA	28 kb	2000	[4]
Japanese encephalitis virus (JEV)	Flaviviridae	+ssRNA	11 kb	2003	[14]
Porcine reproductive and respiratory syndrome virus (PRRSV)	Arteriviridae	+ssRNA	15 kb	2006	[15]
Human coronavirus OC43	Coronaviridae	+ssRNA	31 kb	2006	[16]
Severe acute respiratory syndrome coronavirus (SARS-CoV)	Coronaviridae	+ssRNA	30 kb	2007	[17]
Dengue virus type 1	Flaviviridae	+ssRNA	11 kb	2007	[18]
Bovine viral diarrheal virus (BVDV)	Flaviviridae	+ssRNA	12 kb	2008	[19]
Border disease virus (BDV)	Flaviviridae	+ssRNA	12 kb	2010	[20]
Classical swine fever virus (CSFV)	Flaviviridae	+ssRNA	12 kb	2010	[20]
Respiratory syncytial virus (RSV)	Paramyxoviridae	-ssRNA	15 kb	2012	[21]
Feline infectious peritonitis virus (FIPV)	Coronaviridae	+ssRNA	29 kb	2012	[22]
Middle East respiratory syndrome coronavirus (MERS-CoV)	Coronaviridae	+ssRNA	30 kb	2013	[3]
Dengue virus type 2	Flaviviridae	+ssRNA	11 kb	2014	[23]

## 3. Positive-Strand RNA Virus Biology, Reverse Genetics, and Vaccine Design

Positive-strand RNA viruses have genomes which are infectious upon entry into host cells. Upon entry, host ribosomes translate the viral RNA into one or more polyproteins, which require either host or viral proteases for processing. Although many different reverse genetics platforms have been used to generate positive-strand RNA virus clones, nearly all share the common goal to introduce either sense genomic RNA transcripts directly or cDNA to be transcribed by an included RdRp (Figure 1). Below we describe approaches that have been employed to generate infectious clones of picornaviruses, coronaviruses, and flaviviruses and how these strategies have been and are currently being applied to develop vaccines.

### 3.1. Picornaviruses: Poliovirus and Rhinovirus

Picornaviruses are a family of non-enveloped, positive-strand RNA viruses, which collectively infect a wide range of human and animal hosts. Human picornaviral pathogens cause illnesses, which vary from the common cold to poliomyelitis. The picornavirus virion consists of an icosahedral capsid structure surrounding an RNA genome ranging from approximately 7 to 9 kb in size. Poliovirus and rhinovirus remain two of the most extensively studied picornaviruses to date and have become model systems for the study of RNA virus biology, pathogenesis, and epidemiology. During the 1950’s, extensive efforts began to develop and implement a polio vaccine in the hopes to eradicate the virus since no non-human primate reservoirs are known to exist in nature. The effectiveness of the inactivated (Salk) vaccine and live-attenuated (Sabin) vaccines led to increased efforts to develop and design vaccines for many other human pathogens. International vaccination efforts are aimed at eradicating the disease. The success and employed strategies for implementation of vaccines for poliovirus opened the door to evaluating new approaches and efforts to study and understand RNA virus biology. A major step occurred in 1981 when Racaniello and Baltimore introduced a full-length cDNA clone of poliovirus into a cell and recovered infectious virus [2]. Although initially the mechanism by which a cDNA clone in a plasmid could give rise to productive RNA virus infection was unknown, the cDNA platform approach used by Racaniello and Baltimore was rapidly adapted to develop similar reverse genetics systems for a number of picornaviruses including Coxsackie B virus [24], Rhinovirus [25], Hepatitis A virus [26], Theiler’s virus [27], Foot and Mouth Disease virus [28], Swine Vesicular Disease virus [29], and two Echoviruses [30,31]. Advances in the understanding and engineering of plasmids for gene delivery led to more efficient cDNA-based systems, which represent the primary reverse genetics approaches used for recovery of picornaviruses today. 

No commercially available rhinovirus vaccines exist today despite effective reverse genetics platforms for several serotypes. Rhinoviruses remain the primary cause of the common cold worldwide and have been extensively studied for over 50 years. Yet, the great puzzle of developing a broadly protective rhinovirus vaccine involves solving the overwhelming task of developing an immunogenic platform which can provide protective immunity to the greater than 100 serotypes of rhinovirus that currently circulate in nature [32,33]. The capsid of rhinovirus is comprised of four distinct serotype-specific proteins (VP1, VP2, VP3, and VP4), which are known to be immunogenic and potentially elicit cross-reactive antibodies [32,34,35,36]. Yet, development of broadly-neutralizing rhinovirus protection will likely require the formulation of a polyvalent vaccine which includes incorporation of several serotype variants of the immunogenic capsid proteins [36]. Efforts at designing polyvalent rhinovirus vaccine formulations have yet to provide the broad immunogenic memory necessary to be effective. However, regions of VP0 (precursor of VP2 and VP4) have been shown to be conserved across A and B group rhinoviruses, and combination with a T_H_1 promoting adjuvant induced a cross‑serotype immune response in mice [34]. The discovery of conserved epitopes and recent development of chimeric or new reverse genetics platforms including a mouse model of infection may provide a novel avenue to the efficient display of epitopes from the breadth of rhinovirus diversity [37,38,39].

### 3.2. Coronaviruses

Coronaviruses are enveloped, positive-strand RNA viruses which encode the largest known RNA virus genomes varying in size from 26 to 32 kb [40]. Coronavirus infections in humans are associated with upper and lower respiratory illness ranging in severity from the common cold to severe acute respiratory syndrome (SARS). Over the last 12 years, four new human coronaviruses have been identified including SARS coronavirus and the recent Middle East respiratory syndrome (MERS) coronavirus [41]. Despite a high clinical and economic burden and potential for emerging infectious disease, no commercially available vaccines currently exist. 

There are currently two common platforms that are used for generating full-length infectious coronavirus clones. The first platform involves *in vitro* transcription and capping of a full-length cDNA clone of the coronavirus genome followed by introduction into competent cells by either transfection or electroporation. The coronavirus genome, which is approximately 30 kb in size, is often maintained as fragments in low-copy plasmids. During virus assembly, the fragments are restriction digested and ligated together prior to transcription. This approach has been successfully used to recover clones of many coronaviruses including transmissible gastroenteritis virus (TGEV) [8], murine hepatitis virus (MHV) [10], NL63 [42], SARS coronavirus [9], SARS-like Bat coronaviruses [43], and MERS coronavirus [5]. Another common platform utilizes existing BAC technology. The coronavirus genome is introduced into a BAC construct under the control of a CMV promoter. This approach has been successfully used to recover clones of human viruses OC43 and MERS coronavirus [3,16]. Both of these approaches yield productive infections and high titers of progeny virus. A third approach involving a vaccinia vector platform has also been used successfully for the production of human 229E infectious clones [7].

Efforts to design a coronavirus vaccine have focused on a variety of approaches including development of inactivated virus, live-attenuated virus, and a variety of subunit vaccines. One major focus of current coronavirus vaccine efforts is focusing on ways to create a live-attenuated vaccine strain, which combines existing treatments with a less virulent and more stable virus platform. During the SARS epidemic, patients showed little improvement when treated with ribavirin. Recent studies have shown that coronaviruses are resistant to ribavirin treatment due to the presence of a viral exonuclease (nsp14) with proofreading activity [44,45]. Additionally, deletion of the exonuclease proofreading activity results in a hypermutation phenotype that appears genetically stable and induces protection in murine models [46,47,48]. The recent outbreak of MERS-CoV has triggered a demand for the development of a MERS vaccine [49]. Several approaches are being explored including identifying effective neutralizing antibodies, use of the receptor-binding domain of the spike glycoprotein as a component to induce immunity, and direct changes to recombinant MERS-CoV [3,50,51,52]. The availability of effective reverse genetics platforms for coronaviruses associated with high mortality viruses such as SARS and MERS coronaviruses, and the potential for low cost for development and implementation provide promise for creating an effective vaccine platform. 

### 3.3. Flaviviruses: Yellow Fever, Dengue, and West Nile Viruses

Flaviviruses are small, enveloped, positive-strand RNA viruses that infect a wide range of hosts. Flavivirus genomes are markedly smaller than coronaviruses at approximately 10–12 kb in size, and the transmission of most flaviviruses is dependent upon an arthropod vector (hence their common name, arboviruses). Flavivirus diseases range from asymptomatic to severe neurological disease such as encephalitis, meningitis, and myelitis [53]. Yellow fever virus (YFV), a deadly flavivirus associated with over 30,000 deaths annually (WHO), was identified in 1901 by Walter Reed and was the first human viral pathogen ever discovered [54,55,56]. Outbreaks and disease connected to flaviviruses have stressed the importance of developing reverse genetic platforms and efficacious vaccines.

Similar to picornaviruses, nearly all flavivirus reverse genetics platforms involve either *in vitro* transcription of full-length or ligated cDNA fragments of the genome or use a bacterial artificial chromosome (BAC) platform (Table 1) and are subsequently transfected or electroporated into competent cells. Reverse genetics platforms have been developed for a wide range of flaviviruses including YFV [57,58], Dengue Types 1–4 [59,60,61,62,63,64,65,66], JEV [67], Kunjin virus [68], Tick-borne encephalitis virus (TBEV) [69,70,71], Murray Valley encephalitis virus [72], Langat virus [73], West Nile virus (WNV) [6,74], and Omsk hemorrhagic fever virus [75]. Advances in the recombinant flavivirus approaches have been instrumental in new vaccine design efforts. 

Some of the first vaccine efforts were directed at generating inactivated or live-attenuated strains for vaccinations against flaviviruses. In 1937, Max Theiler developed a safe YFV live-attenuated vaccine called 17D through the use of a serial passaged virus originally isolated from an African patient [76]. The vaccine composition used today remains largely the same as that first developed over 70 years ago and provides protective immunity for over 30 years [77,78,79]. JEV was first isolated and studied in the 1930’s and an inactivated vaccine derived from a mouse brain was first developed in Japan in 1954 [53,80]. The current formulations of the JEV vaccines include inactivated Beijing-1 strain or live‑attenuated strains, which elicit greater immunogenicity and broader protection than the original Nakayama strain [53]. Despite the success of vaccine development for YFV and JEV, unique challenges have been presented in developing vaccines to some well-known flaviviruses (such as dengue virus) and new emerging flaviviruses (such as WNV). 

Dengue virus is endemic to tropical and subtropical locations worldwide. To date, five distinct serotypes of dengue virus are known, including a new serotype identified in 2013 [53,81]. Infection with one serotype increases the severity of disease upon a secondary infection with a different serotype [82]. Consequently, any dengue virus vaccine must either provide protection to all extent serotypes to prevent priming for increased disease by a heterotypic infection or remove the immunogenic components of the virus that cause increased disease severity upon heterotypic infections. Efforts to develop stable chimeric platforms for the development of dengue virus vaccines have recently been focused on expressing dengue virus surface proteins in chimeric viruses with other more stable flaviviruses such as YFV [83,84,85]. One vaccine candidate is a tetravalent vaccine from Sanofi Pasteur, which involves the formation of a chimeric YFV strain 17D virus containing the prM/E genes of each dengue serotype [85]. This approach has also recently been adapted to develop similar vaccine candidates to the recently identified WNV [85,86]. Despite several commercially available WNV vaccines for veterinary purposes, there remains no approved WNV vaccine for humans. The future licensure of dengue and WNV vaccines will likely continue to focus on development of live‑attenuated or inactivated virus models of vaccination due to the high immunogenicity of flavivirus infections.

## 4. Negative-Strand RNA Virus Biology, Reverse Genetics, and Vaccine Design

Negative-strand RNA virus genomes and antigenomes cannot act as an mRNA. To be a substrate of RdRp, they must be encapsidated with nucleocapsid and form ribonucleoprotein complexes (RNPs). The antigenomes of negative-strand RNA viruses are introduced either as a linearized cDNA or as a plasmid under a T7 promoter and are normally co-transfected with one or more plasmids encoding the nucleocapsid and replicase machinery. Due to these biological requirements to initiate infection, development of negative-strand RNA virus reverse genetics systems has been slower than positive‑strand virus systems. Initial recovery efforts to generate infectious clones involved recovery of viruses using helper viruses, which could supply the viral genes and proteins necessary for replication. However, these approaches made it difficult to isolate the mutants of interest. The first recovery of a negative‑strand RNA virus completely from cDNA was achieved in 1994 for rabies virus [87,88]. Despite the biological limitations and challenges to working with negative-strand RNA viruses, several effective negative-strand RNA virus vaccines have been successfully introduced and many more are currently in varying phases of clinical trials. Below we describe approaches that have been employed to generate infectious clones and vaccines in paramyxoviruses and orthomyxoviruses.

### 4.1. Paramyxoviruses: Measles Virus and Respiratory Syncytial Viruses

Paramyxoviruses are enveloped, negative-sense, single-strand RNA viruses which are responsible for a variety of human and animal diseases. Human paramyxoviruses have been identified which are responsible for diseases including measles, mumps, pneumonia, and the common cold. Paramyxoviruses carry a single copy of their genome, which is typically 15 to 19 kb in length. Like other negative-strand RNA viruses, paramyxoviruses must incorporate their replication machinery, including the RDRP, into the virion during assembly. Paramyxovirus reverse genetic systems employ very similar mechanisms. First, the full-length genome or antigenome and helper plasmids expressing nucleocapsid and polymerase proteins are cloned as cDNA and are under transcriptional control by a promoter such as T7 RNA polymerase. The plasmids are co-transfected into permissive cell lines. Earlier reverse genetics systems utilized a co-infection approach with a vaccinia virus expressing T7, however most modern reverse genetics systems use a T7 cell line or transfect a T7 plasmid. Recently, the first BAC-based reverse genetics system for a negative-strand RNA virus was developed for respiratory syncytial virus (RSV) [21]. Reverse genetics systems for many paramyxoviruses have been generated including measles virus [89], mumps virus [90], Hendra virus [91], Nipah virus [92], RSV [21,93]. Despite the availability of reverse genetics systems, development of paramyxovirus vaccines has been met with variable success. 

The first paramyxovirus vaccine was developed during the 1950’s. John Enders was able to develop a cultivation system for measles virus and cultured an attenuated measles virus called the Edmonston strain (named after the child from which it was isolated). The Edmonston strain, though initially under‑attenuated, was later adapted and led to the successfully license of a measles vaccine in 1963 [94]. Maurice Hilleman was able to build on the success of the live-attenuated measles vaccine and cultured and adapted by passage in fertilized hen’s eggs, a strain of mumps called Jeryl Lynn (named after his daughter, from whom it was isolated) [95]. Hilleman was later instrumental in the development of the MMR vaccine combining live-attenuated strains of measles, mumps, rubella (Wistar RA 27/3 strain), which was first licensed for use in 1971 [96]. The early success of the measles and mumps vaccines prompted renewed efforts to develop vaccines to other pathogenic RNA viruses. One of the greatest challenges to viral vaccine design remains the development of a vaccine for respiratory syncytial virus (RSV). 

Respiratory syncytial virus (RSV) was first isolated in 1955 from a chimpanzee displaying upper respiratory illness [97]. Since its identification, RSV has become recognized as the leading cause of infant mortality by a virus worldwide [98,99]. In the United States alone, RSV upper and lower respiratory infections have led to over 100,000 hospitalizations annually [98,99,100]. Despite a high clinical burden, no licensed RSV vaccines are available and current treatments are cost prohibitive while only providing passive immunity by administration of prophylactic antibodies [101,102,103]. The most susceptible population for RSV infection is young infants [98,99,100]. Consequently, the ideal RSV vaccine must be immunogenic, genetically stable, and safe for vaccination in infants. However, an early tragic failure during the 1960’s of a formalin-inactivated RSV vaccine has dampened efforts to develop and implement new vaccines [104,105]. To date, the most clinically advanced RSV vaccine candidates have been live-attenuated viruses developed through virus passage and reconstitution by reverse genetics [106,107,108]. One of the more promising of these candidates to date has been MEDI‑559, which includes many introduced mutations which render the virus temperature-sensitive and provides some level of protection [106,107]. However, these candidates have not been able to achieve the level of protection and genetic stability necessary for implementation [109]. Ongoing studies continue to evaluate new targets for attenuation; however successful development of an RSV vaccine will require finding the proper balance of attenuation and immunogenicity.

### 4.2. Orthomyxoviruses: Influenza virus

Orthomyxoviruses are enveloped, negative-sense RNA viruses whose genomes consist of multiple linear segments and are approximately 12 to 15 kb in size. Similar to paramyxoviruses, many orthomyxoviruses cause respiratory illnesses in humans, most notable is influenza (flu). A key hallmark of orthoymyxovirus evolution is the reassortment of the virus genomes through co-infection of a cell. In order to be transcriptionally active, influenza viruses require a functionally active viral ribonucleoprotein complex (RNP) which consists of the viral genomic RNA, nucleoprotein (NP), and the viral RdRp (comprised of PB1, PB2, and PA proteins) [110]. The first reverse genetics approaches developed for orthomyxoviruses were generated for influenza A virus (IAV), however these systems utilized helper viruses that had to be selected against to recover recombinants [111,112]. The first helper virus-free systems were developed around the turn of the millennium [113,114]. These first systems required the co-transfection of four or more plasmids under the control of a Pol II promoter as well as eight plasmids expressing the eight viral RNA segments. The number of plasmids co‑transfected for recovery varied greatly depending upon the number of viral genomic segments and the organization of the helper protein constructs, ranging from 10 (which expressed two of the helper proteins on each of two plasmids using the Pol II promoter and an IRES) to as many as 17 [113,114,115,116,117,118]. However, recent advances in the reverse genetics platforms have reduced the number of total plasmids needed to 8 or less using a bidirectional expression system [11,12]. In this system, a human Pol I promoter coupled with either a murine PolI terminator drives viral RNA synthesis and a CMV Pol II promoter is responsible for viral mRNA synthesis [11]. This improved production of IAV, however, generation of recombinant virus for vaccines is limited to a select number of mammalian cell lines such as African green monkey kidney epithelial cells (Vero) or Madin Darby canine kidney (MDCK) cells [119,120]. However, these cell lines had limited transfectability and differences in Pol I and Pol II compatibility have hindered the efficacy of these uni- and bidirectional approaches. Incorporation of species-specific polymerase promoters has provided improved efficacy of recovery in several of these cell lines [119,120]. A recent advancement has been the combination of up to 8 Pol I driven IAV genes on a single plasmid and up to 3 Pol II drive genes on an additional plasmid, this approach improves the probability of a single cell receiving all necessary plasmids during recovery [121]. The availability of applicable reverse genetics systems for studying and identifying the structure and function of influenza proteins has revolutionized influenza vaccination strategies. 

For many years, the trivalent vaccine that is currently provided consisted of three separated strains (2 A strains and 1 B strain), which were selected based on the WHO recommendations prior to the next flu season. However, these vaccines were made using either live-attenuated (through cold-adaptation) or inactivated virus grown in fertilized chicken eggs. Next generation vaccination strategies include tetravalent or quadrivalent vaccines, which may be grown in animal cell cultures (rather than chicken eggs) or virus-like particles (VLPs) in cultures of *S. frugiperda* insect (Sf9) cells [122,123,124,125,126,127,128,129]. In 2013, the FDA approved a seasonal influenza vaccine comprised of purified HA proteins prepared using a baculovirus-expression system [130]. These recombinant vaccines were successfully used to vaccinate individuals between 18 and 49 years of age and represents a major step in influenza vaccine design and implementation because this system reduces production time compared to the conventional egg-based approach [131]. 

## 5. Synthetic Biology and the Future of RNA Virus Vaccine Design

### 5.1. Limitations and Challenges to the Application of Reverse Genetics to Vaccine Design

A major frontier of synthetic biology is the development of new vaccines and therapeutics and the improvement of the implementation and efficacy of those already on the market. One major complication to the successful implementation of vaccines currently is the limitations in time between vaccine design and production. Influenza viruses require seasonal vaccinations and determination of the proper formulations leaves little time for development and implementation. New advances in reverse genetics technology are reducing the potential time of recovery and production from months to weeks. For instance, Dormitzer *et al.* have developed an improved platform approach and demonstrated that with current reverse genetics technology, it is feasible to generate a recombinant influenza virus from new HA and NA sequences within 5 days [132]. As new emerging viruses continue to appear and pandemics continue to occur, rapid approaches to recover and adapt viruses will be instrumental in the public health response. Development of common chimeric reverse genetic platforms for rapid cloning and expression of surface antigens from emergent pathogens will help increase the efficacy and timing of delivery during epidemic and pandemic outbreaks.

One key limitation to the development of effective live-attenuated vaccines is genetic stability. RNA viruses generally exhibit high mutation rates due to decreased fidelity of the RDRP. Yet, recent studies have shown that codon-usage bias can be used to alter the translation and consequently, replication of viruses. Several groups have shown that substituting non-preferred codons based on host cell codon usage bias, a process referred to as codon-deoptimization, into the genome of poliovirus resulted in reduction of plaque areas and virus yields [133,134]. By changing codons rather than amino acids, the amount of viral protein may be modulated without impairment in its function. More importantly, the potential for reversion is greatly limited due to the sheer number of codon changes introduced into the coding sequence. This approach represents a promising new avenue to develop attenuated, but genetically stable vaccine platforms.

### 5.2. Current and Future Directions to Vaccine Design

Despite the presence of reverse genetics systems for many human pathogens, not all have resulted in successful vaccine platforms. Advances in biotechnology and key discoveries have led to novel reverse genetic approaches, which may be employed in new generation vaccines. Structural vaccinology or structure-based antigen design has become a common practice for optimizing antigens for display in vaccines [135]. As mentioned previously, RSV remains a key hurdle to reducing viral childhood morbidity. Since the discovery and implementation of palivizumab, currently the only licensed prophalytic inhibitory measure to RSV infection, considerable energy has been devoted to evaluating the antigenic sites of the RSV fusion (F) protein and optimizing F expression constructs for higher immunogenicity [105]. Current structural vaccinology efforts to evaluate the pre-fusion and post-fusion antigenic forms of RSV F protein have led to the induction and identification of potent neutralizing antibodies with higher neutralizing potencies than palivizumab [136,137]. The availability of new structures and sequences has made predictive structural modeling and structure-based antigen design viable options for renewed efforts at design of vaccines, for which no current vaccines exist.

Advances in biotechnology have shaped the field of virology as much as any other field of science. The capacity to engineer reverse genetics platforms for the study and manipulation of RNA virus genomes has revolutionized the field of vaccine design. We have described here RNA virus reverse genetic systems and past and current efforts to develop vaccines to provide immunity to several human RNA virus pathogens. The incorporation of new advances in reverse genetics technology, adjuvants, non-human models of infection, and surveillance will continue to drive the development of next generation vaccines to pathogens for which we already have vaccination strategies as well as those that we currently do not. Ongoing changes in human demographics and accessibility to health care are likely to modify cost-benefit analyses and provoke allocation of new resources for scientific study and development of vaccines. Despite the ongoing evolution of vaccine design and implementation, the hallmarks of an effective viral vaccine will remain the same: high efficacy, safety, and stability.
